# Assessment of Polyhydroxybutyrate Production by Cyanobacteria Strains Isolated from Environmental Water Sources Using a Secondary Effluent

**DOI:** 10.1021/acsestwater.5c00677

**Published:** 2025-11-07

**Authors:** Artai Lage, Esther Berrendero Gómez, Laura García-Abad, Cristina Martínez-Gutiérrez, Joan Garcia, Eva Gonzalez-Flo

**Affiliations:** 1 GEMMA-Group of Environmental Engineering and Microbiology, Department of Civil and Environmental Engineering, Escola d’Enginyeria de Barcelona Est (EEBE), Universitat Politècnica de Catalunya·BarcelonaTech, Av. Eduard Maristany 16, Building C5.1, Barcelona E-08019, Spain; 2 Institute for Agro-Food and Agro-Environmental Research and Innovation, Department of Applied Biology, 16753 Miguel Hernández University of Elche (CIAGRO-UMH), Elche 03202, Spain; 3 GEMMA-Group of Environmental Engineering and Microbiology, Department of Civil and Environmental Engineering, Universitat Politècnica de Catalunya·BarcelonaTech, c/Jordi Girona 1-3, Building D1, Barcelona E-08034, Spain

**Keywords:** PHB, BG11 media, secondary effluent

## Abstract

Growing concern over plastic pollution has intensified research on biodegradable alternatives, such as polyhydroxybutyrate (PHB), a biopolymer produced by cyanobacteria. Despite their sustainability advantages, photoautotrophic PHB production remains limited, and cultivation strategies need optimization. In this study, five cyanobacterial strains were isolated from environmental microbiome cultures to evaluate their PHB production potential. The goal was to identify the most productive strains and optimal conditions for polymer synthesis. Cultures were grown in modified BG11 media (without nitrogen, phosphorus, or inorganic carbon) and in a secondary effluent from treated urban wastewater, both supplemented with acetate (0, 0.6, or 4 g/L) and incubated for 7 days in darkness. The biomass remained stable in most strains but declined to 0.28 g/L in the secondary effluent, except for one *Leptolyngbya* sp. strain that increased the biomass with acetate. The highest PHB yield per acetate consumed was achieved by *Synechocystis* sp. from an agricultural pond, reaching 3.1% dry cell weight in modified BG11 with 0.6 g/L acetate. In the secondary effluent, the maximum PHB content reached 2.9% in another *Leptolyngbya* sp. strain with 4 g/L acetate. These findings highlight strain-specific responses and the potential of wastewater-based cultivation for sustainable bioplastic production.

## Graphical abstract

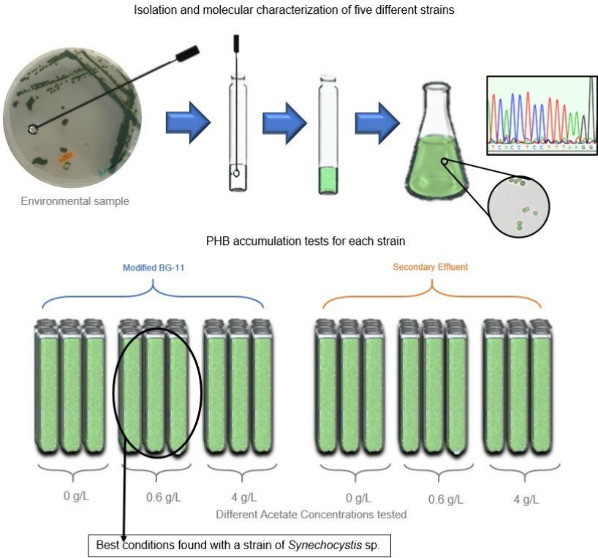

## 1 Introduction

The increasing concern for environmental plastic pollution has led to the search for sustainable alternatives based on bioplastic products, thus avoiding petroleum derivatives. Among several alternatives, bioplastics derived from cyanobacteria are a promising solution. These microorganisms are capable of producing polyhydroxyalkanoates (PHA), mostly in the form of polyhydroxybutyrate (PHB).^1^

Cyanobacteria obtain energy by oxygenic photosynthesis and use CO_2_ as the main carbon source, even though most strains may perform a heterotrophic metabolism.^2,3^ Actually, some basic organic compounds, such as acetate, can serve as PHB production promoters. In fact, acetate is a precursor for acetyl-CoA synthesis, which is necessary for starting the PHB synthesis pathway.^4^

PHB is a fully biodegradable polymer with similar properties to polypropylene.^5^ However, a significant challenge is its higher costs with the current production processes in comparison to petroleum-based products.^6^ Hence, there is a rising interest in finding production process alternatives that could lower PHB costs. Cyanobacteria cultures are indeed of main interest over the common employed heterotrophic bacteria production processes that require high amounts of organic feedstocks and energy for cultures’ aeration, making the whole process costly and noncompetitive.^7^

Technologies based on cyanobacteria are highly relevant because their growth and maintenance do not require expensive organic feedstocks.^8^ Moreover, these microorganisms are starting to get an important role in the field of biotechnology because they are microorganisms with a great evolutionary background, thus allowing them to adapt to many different environments, even to extreme habitats.^9^ In consequence, these organisms are great candidates for being cultured in complex variable media such as treated and untreated wastewaters.^10^ Their inorganic nutrient requirements allow them to grow easily in wastewaters that are rich in dissolved nitrogen and phosphorus such as secondary effluents.^11^ In fact, in the past years, there have been many published studies on the potential application of these microorganisms for wastewater treatment and bioremediation.^12,13^ Those treatments do not require oxygen for aeration because the photosynthetic activity of cyanobacteria supplies oxygen for the degradation of contaminants.^14^

Despite all the advantages that cyanobacteria display, the PHB production rates obtained up to date are insufficient for the development of full-scale productive processes.^15^ Previous research has mainly focused on model cyanobacteria strains such as *Synechocystis* sp. and *Synechococcus* sp., reporting PHB contents that, although relevant, still fall short when compared to heterotrophic bacterial systems.^16^ PHB production by cyanobacteria has been widely described in the literature. For instance, cultures of *Synechocystis* sp. have been reported to achieve PHB contents of approximately 11% dry cell weight (dcw) under mixotrophic conditions with acetate supplementation.^17^ Even higher values, up to 25% PHB in dcw, were reported when *Synechocystis* was cultivated in wastewater-based systems.^18^ Other relevant studies include work with *Leptolyngbya boryana*, which has been shown to accumulate up to 32% PHB of dcw under nutrient-depleted BG11 medium supplemented with 2 g/L acetate.^19^

In contrast, reports for *Synechococcus* sp. show highly variable PHB productivities depending on the strain and culture conditions, ranging from less than 1% dcw^20^ to as high as 30%.^21^ These contrasting reports highlight the importance of screening new environmental strain variants from these genera to identify candidates with a superior PHB productivity. More recently, studies have explored the use of environmental isolates with promising tolerance to stress conditions and higher PHB accumulation potential.^22^ Nonetheless, a systematic comparison of multiple environmental cyanobacteria strains, especially in the context of wastewater-based cultivation, remains limited.

Thus, there is growing interest in finding new strains of cyanobacteria with a higher potential for PHB accumulation capacity. In this sense, the novelty of this study lies in combining the screening of environmentally isolated cyanobacteria strains for PHB production with the evaluation of their performance in secondary effluents from treated wastewater. This dual approach addresses both the urgent need for cost-effective PHB production and the valorization of wastewater as a growth medium, contributing to the development of integrated biorefinery strategies. In line with this, the aim of the present study was to evaluate five cyanobacteria strains (two strains belonging to the *Synechocystis* sp. genus, two strains belonging to the *Leptolyngbya* sp. genus, and one belonging to the *Synechococcus* sp. genus) isolated from environmental samples for their PHB production potential in order to be implemented into a pilot test in a higher scale.

Although these strains belong to species that have previously been reported for their capacity to synthesize PHB,^19^ it is important to note that, unlike strains commonly obtained from algal culture collections, the isolates in this work were directly recovered from the environment. This distinction may confer unique genetic traits or adaptive characteristics, potentially enhancing their PHB productivity beyond that of previously studied counterparts. In addition, the viability of using a secondary effluent from treated wastewaters was tested for PHB production. In this study, a two-stage cultivation strategy was adopted in which cyanobacteria were first grown under photoautotrophic conditions to support biomass accumulation, followed by PHB induction under heterotrophic conditions. This approach leverages the cost-effectiveness of photoautotrophic growth, which relies solely on light, CO_2_, and inorganic nutrients, thereby reducing the expenses associated with organic substrates and oxygen aeration typically required in heterotrophic systems. At the same time, the heterotrophic phase enables enhanced PHB accumulation by redirecting cellular metabolism toward polymer synthesis. The integration of both modes of metabolism represents a resource-efficient strategy that balances growth sustainability with maximized PHB productivity.

It is hypothesized that different cyanobacterial isolates will display distinct PHB accumulation capacities, thereby justifying a screening approach to identify strains with the potential for scalable production. Furthermore, it is expected that secondary effluents, despite their high nitrogen content, can serve as a viable medium for PHB synthesis by inducing cellular stress responses that promote polymer accumulation.

## 2 Materials and Methods

### 2.1 Sample Procurement and Strain Isolation

The five different strains of unicellular and filamentous cyanobacteria were isolated from existing cultures of microbiomes that were obtained from environmental samples^23,24^ (Table S1, Supporting Information). Strains were isolated using a combination of plate colony isolation methods and a single-cell isolation protocol.^25^ All the isolation process was performed under sterile conditions, in a horizontal laminar flow hood (Cruma, CRLF1). The whole media and instruments used were previously autoclaved.

First, 100 μL of each culture obtained from environmental samples was inoculated into a 40 mL BG11-agar plate to spot the different morphologies corresponding presumably to the different strains (Figure 1A). Individual colony types were then spread with sterile loops into separate BG11-agar plates, as shown in Figure 1B until clear single colonies of uniform morphology were reached on each plate. Normally, this process took about four rounds of plating per strain until pure cultures were obtained with a single morphology. After obtaining colonies with a uniform morphology, single colonies were collected with a sterile loop for unicellular strains (Figure 1C1) and by the hook-dragging method^26^ for filamentous strains (Figure 1C2).

**Figure 1:**
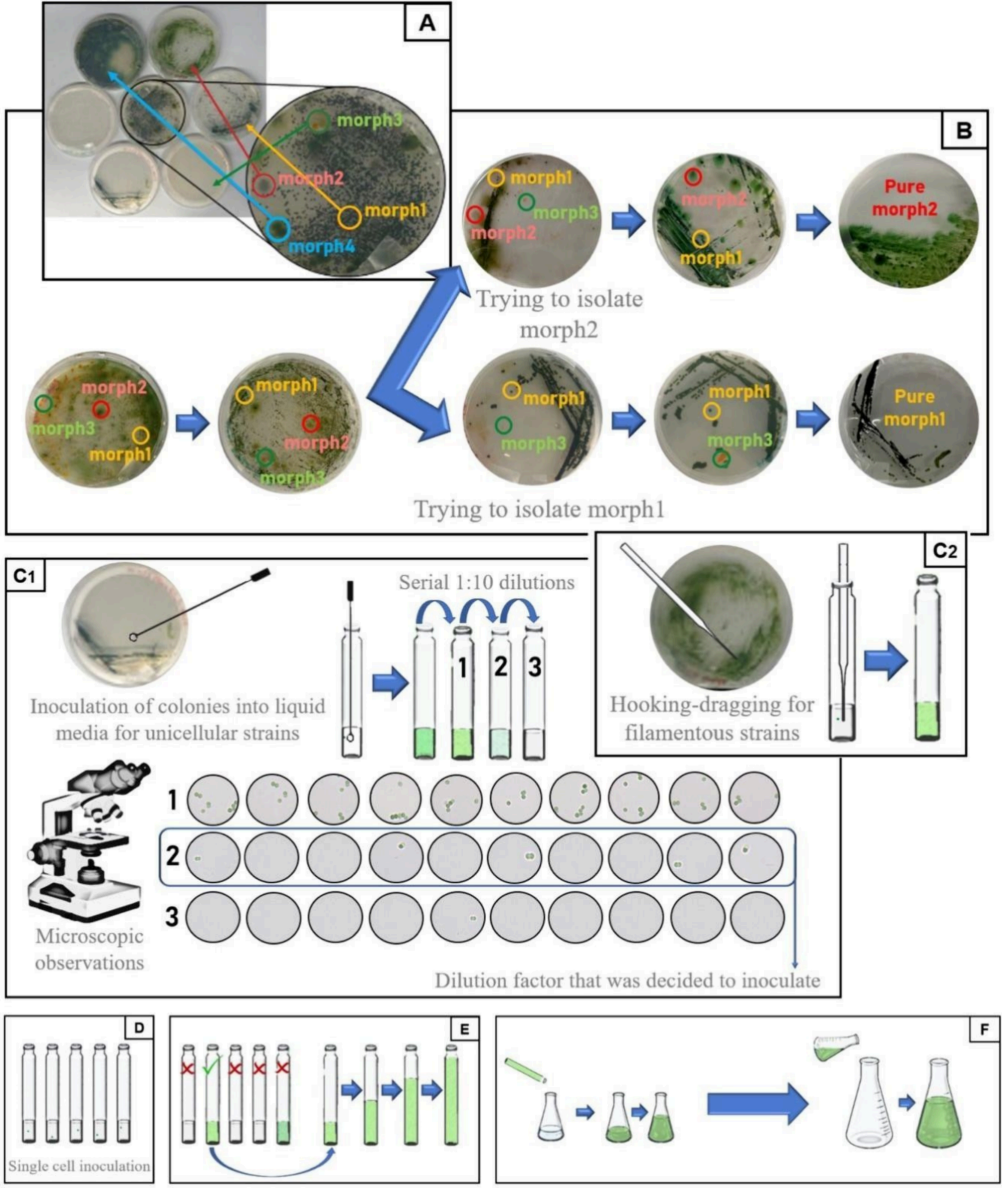
Isolation and purification methodology carried out for each strain tested in this research. (A) Picture of the agar Petri dishes containing all strains from a microbiome culture sample and the resulting agar plates harvested with each of the different strains. (B) Plating process until obtaining pure colonies of each morphology (morph). (C1) Single-cell isolation by the dilution technique. Started picking up a single colony with a sterile loop and inoculation into BG11 liquid media. Then, dilutions of the liquid inoculation of the colony were observed by microscopic observations from a 10 μL drop. (C2) Hooking-dragging method. (D) Inoculation of 10 μL drops of the corresponding dilution into fresh BG11 media for unicellular strains and inoculation of single filaments for filamentous strains. (E) Scheme showing the vials that were discarded because of lack growth or overgrowth due to high cellular initial inoculation and scaling-up of the vials where growth was appreciated after 2 weeks. (F) Inoculation and scaling-up in Erlenmeyer flasks.

For unicellular strains, the colonies that were picked up with the sterile loop were inoculated into a fresh BG11 liquid medium for performing serial dilutions. These serial dilutions were checked under the microscope (Figure 1C1). Once a single cell was detected in at least five out of 10 microscopic observations in a 10 μL drop, it was decided to inoculate that volume of the corresponding dilution into vials containing 2 mL of fresh BG11 liquid medium (Figure 1D). When more than a cell or just one cell was observed in less than five out of 10 observations, it was decided not to inoculate with that particular dilution.

Regarding filamentous cyanobacteria, colonies were left to grow in BG11-agar plates and isolated under a microscope (Figure 1C2). Single filaments were manually picked with a hook made from a Pasteur pipet using fire to bend the desired shape.^26^ Then, the single filaments were transferred into vials containing 2 mL of fresh BG11 liquid medium (Figure 1D).

Vials inoculated with both of the aforementioned methods were incubated with five replicas per strain to ensure that at least one vial could develop a population from a single cell or filament (Figure 1D). The vials where growth was not noticed were discarded after a month. Conversely, the vials in which growth was appreciated too fast in less than a week were also discarded, on the grounds of probably starting the population from a big group of cells rather than a single one (Figure 1E). For the ones where growth was observed after 2 weeks (slight green turbidity or small filaments forming in the vials), the initial 2 mL volume was escalated with BG11 in a 1:4 fashion every week until a total volume of 10 mL was reached (Figure 1E) and then transferred in 100 mL Erlenmeyer flasks (Figure 1F). Those flasks were escalated until the content reached 1 L flasks, resulting in two flasks of 1 L per strain. Those flasks were placed in an incubator (Memmert, BioLab) and maintained at the conditions briefly explained in Section 2.3 Culture Maintenance.

### 2.2 Morphology and Molecular Identification

Morphological observations of isolated strains were made using an Olympus BX 51 (Olympus Corp., Tokyo, Japan) photomicroscope equipped with Nomarski DIC optics and an Olympus DP71 digital camera. The identification of strains was done according to traditional features described by Komárek et al.^27^ For the molecular analyses, several single cells and filaments were isolated under sterile conditions using an isolation technique as described by Mareš et al.^28^ A segment of the rRNA operon, including the 16S rRNA gene and the 16S-23S internal transcribed spacer (ITS) region (1800–2000 bp), was amplified using the primers pA^29^ and KP.591R^30^ under the conditions described by Gkelis et al.^31^ The PCR products were purified by gel electrophoresis and then cloned using the pGEM-T Easy Vector Systems (Promega Corp., Madison, WI, USA).^32^ The plasmids containing inserts were purified from *Escherichia coli* using a Nucleospin plasmid kit (MACHEREY-NAGEL, Düren, Germany) and then sent for STAB Vida (Caparica, Portugal) for sequencing. Nucleotide sequences obtained were processed to correct errors using BioEdit software version 7.7.1.^33^

The complete sequences were compared with the information available in the National Center for Biotechnology Information (NCBI) database using the BLAST algorithm (http://www.ncbi.nlm.nih.gov/BLAST).

Sequences obtained were submitted to the NCBI GenBank database.

### 2.3 Culture Maintenance

1 L culture flasks were grown in an incubator (Memmert, BioLab) at 30 °C under light:dark cycles of 15:9 h of white light at 300 μmol/(m^2^ s) and agitated at 250 rpm in BG11 liquid media with a sodium bicarbonate modification of 1.75 g NaHCO_3_/L to maintain pH values between 8 and 9.^24^ Once a week, a purge of 300 mL was done to add the previously described BG11 fresh media.

### 2.4 Biomass Quantification

In this study in order to have a fast method for biomass, it was measured from a correlation between turbidity (expressed as NTU, nephelometric turbidity units) and VSS (volatile suspended solids), as described in the American Public Health Association (2012).^34^ To create the correlation for each individual strain, both turbidity (HI93703, HANNA Instruments) and VSS were measured during a week of constant growth. That way, the calibration curves in the Supporting Information were obtained (Figure S2, Supporting Information). Note that for filamentous strains, this correlation could not be done due to the formation of flocs that hindered turbidity reading's reliability. As a result, VSS was measured directly.

### 2.5 Experimental Setup

In order to evaluate the capacity of each strain for PHB production, three different acetate conditions were tested in two different types of media: (i) modified BG11 with no nitrogen, phosphorus, and inorganic carbon source and (ii) the secondary effluent coming from an urban wastewater treatment plant near our laboratory premises. In both cases, biomass was previously grown in the same BG11 media under photoautotrophic conditions. The modified BG11 was elaborately depleted in nutrients in order to induce PHB.^35^ The secondary effluent had nutrients that were analyzed following the methodology in 4500-N, 4500-NH3, and 4500-P.^34^ PHB accumulation was also evaluated using the secondary effluent, despite its high nitrogen content, in order to assess whether this type of medium induces cellular stress and determine if nitrogen availability influences PHB synthesis under conditions lacking other nutrients typically supplied in standard media for photoautotrophic cells, such as BG11. The same stock of secondary effluent was used to perform the tests for all strains; therefore, they were evaluated under identical nutrient concentration conditions.

The whole combination of six conditions with three replicates per condition were tested in 50 mL tubes inoculated for each different strain. All tubes were set at an initial biomass concentration of 0.4 gVSS/L for all. The starting biomass for inoculation came from the stocks of each strain that were kept on two Erlenmeyer flasks of 1 L inside the incubator (Memmert, BioLab). All the tubes were left for a week under darkness to ensure anoxic conditions in order to enhance the PHB synthesis.^36^ Tubes were mixed by bubbling nitrogen gas. After 1 week, pH, electrical conductivity, VSS, and PHB were measured.

### 2.6 PHB Analysis

PHB had to be extracted from the biomass on the grounds that this polymer is accumulated within the cell cytosol. Hence, for PHB extraction, cultures had to be centrifuged at 15,000 rpm for 4.5 min. Once the pellet from the centrifugation was obtained, the supernatant was removed and the Eppendorf tube containing the biomass was kept in the freezer at −20 °C for at least 24 h. Then, all tubes were frozen for 24 h at −80 °C prior to overnight lyophilization. In order to extract the PHB from that biomass, between 3.0 and 3.5 mg of lyophilized biomass was weighted and the following protocol was followed.

First, the extracting reagents had to be prepared. 100 mg of benzoic acid (99.5%) was diluted in 200 mL of a chloroform solution (150 ppm stabilized with amylene). A solution of known concentration of PHB was made for the sake of having a calibration curve for the sample lectures. Thus, 77 mg of PHB was diluted in 50 mL of the previously made acidic solution of chloroform. In addition, an acidic methanol solution with sulfuric acid had to be made. 20 mL of sulfuric acid (98%) was poured into 20 mL of a methanol solution (99.8%). Given that this reaction is strongly exothermic, it was needed to be done in ice. Then, methanol was poured until a volume of 100 mL was reached.

With these reagents, PHB extraction could be achieved. To each sample of lyophilized biomass were added 1 mL of the acidic chloroform solution and 1 mL of the acidic methanol solution. Then, all samples were digested for 5 h at 100 °C. After digestion, all samples were kept in ice for 30 min. Thereafter, 1 mL of distilled water was added and mixed for 1 min until solvents were separated by density. The bottom chloroform phase is where PHB was remaining. 850 μL from the bottom phase was taken and stored in a hermetic closed tube for its lecture. PHB was determined by gas chromatography (7820A, Agilent Technologies, USA). As a result, the values of PHB obtained were expressed in percentages of PHB in dry cellular weight (dcw).

### 2.7 Statistical Analysis

All results were statically analyzed by a multifactorial ANOVA using JMP software. There were three replicates for the 30 different conditions tested.

## 3 Results and Discussion

### 3.1 Morphological and Molecular Identification of Strains

Following the methodology of single-cell isolation, it was possible to obtain the five different strains of cyanobacteria, which are described below (Figure 2). All strains are named after the genera in which they belong followed by the initials of the place where they were isolated from. AP stands for agricultural pond, UP urban pond, R river, and BS the river Besòs shore.

**Figure 2:**
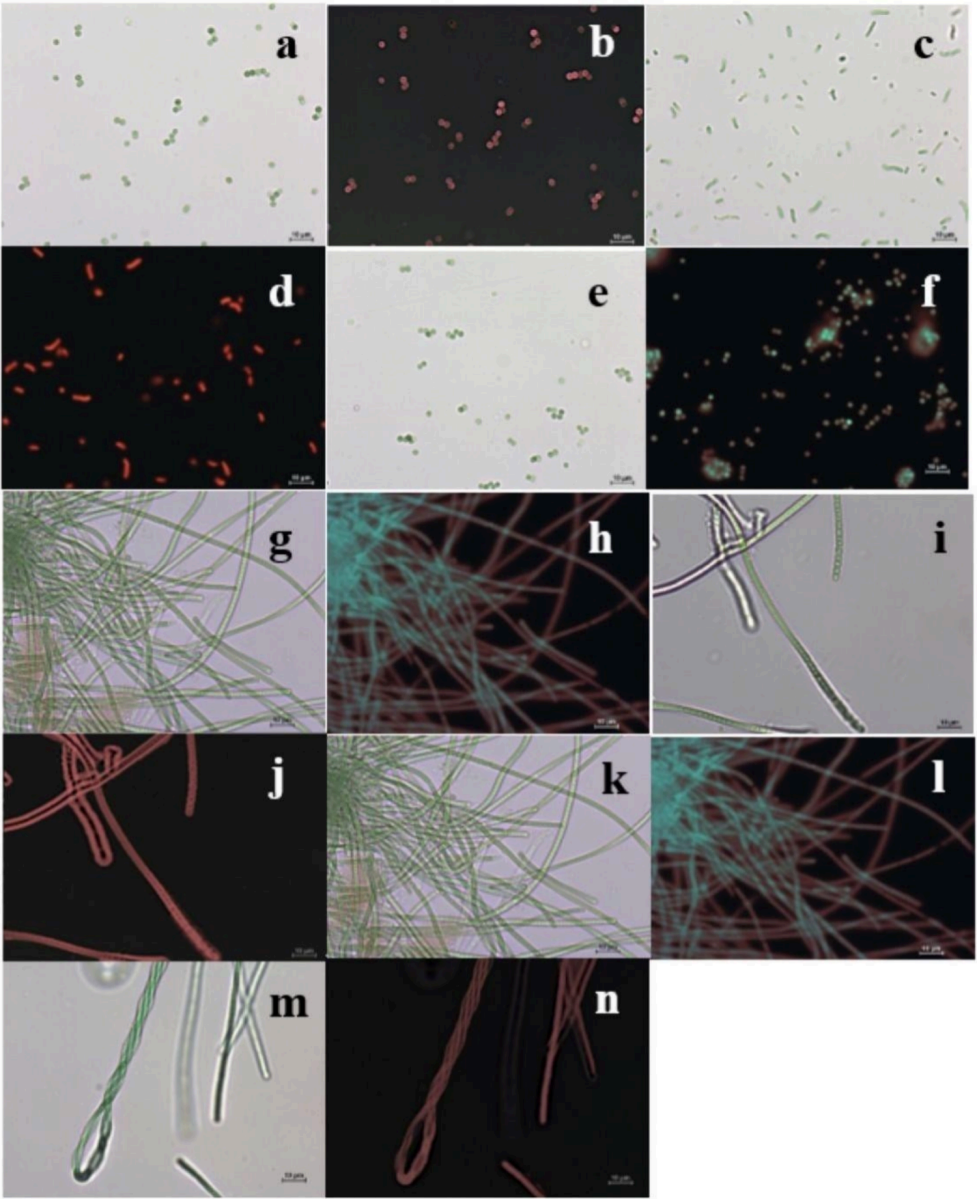
Images of the strains under a light bright microscope and their corresponding pictures under a fluorescence microscope (red-dark images). (a, b) *Synechocystis* sp. AP. (c, d) *Synechococcus* sp. AP. (e, f) *Synechocystis* sp. UP. (g–j) *Leptolyngbya* sp. R. (k–n) *Leptolyngbya* sp. BS. All images at a 400× magnification. Pictures taken with a camera (Fi2, Nikon, Japan) and a fluorescence microscope (Eclipse E200, Nikon, Japan) using NIS-Elements Viewer software.

#### 3.1.1 *Synechocystis* sp. AP

This strain is a unicellular cyanobacteria characterized by having solitary cells in general or agglomerated without common mucilage. The cell form is spherical or slightly widely oval before division, being morphologically identified as species belonging to the genus *Synechocystis*. The partial 16S rRNA sequence from this strain (1482 pb) was 99.00%, identical to other *Synechocystis* species tested, for example, *Synechocystis* sp. CACIAM 05 (accession no. CP019225), isolated from a freshwater lake from Brazil; *Synechocystis* sp. PCC 6714 (CP007542) from freshwater in California (USA); and *Synechocystis* sp. YACCYB312 (MH683739) that was from China. These data confirmed its identification as *Synechocystis* sp.

#### 3.1.2 *Synechococcus* sp. AP

This strain is observed in the form of solitary cells or grouped in irregular clusters but not forming mucilaginous colonies. Cells are long oval or cylindrical and usually pale or olive green containing granules in the terminal parts of the cell. The partial 16S rRNA sequence from this strain (1487 pb) after comparing with the GenBank database was 99% identical to the ribosomal rRNA sequence from *Synechococcus* sp. PCC 6312 (accession no. CP003558), from freshwater in California (USA) described in the Pasteur collection, and 99% identical to *Synechococcus* sp. PCC 6603 (accession no. MK484711) also isolated from a pond in California (USA).

#### 3.1.3 *Synechocystis* sp. UP

The morphological description of this strain is completely the same as that of the aforementioned *Synechocystis* sp. AP. The complete 16S rRNA sequence from this strain (1480 bp) showed 99.93% identity with *Synechocystis* sp. CACIAM 05 (accession no. CP019225.1), which was isolated from a freshwater lake in Brazil. This strain also presented 99.78% identity with an uncultured *Synechocystis* sp. clone, OTU10 (accession no. MN493570.1) cultivated in our laboratory. These findings support its classification within the genus *Synechocystis.*

#### 3.1.4 *Leptolyngbya* sp. R

Through microscopic observations, the morphology of this strain is as follows: long filaments formed of a thin sheath, hyaline, and homogeneous; solitary or joined in thin biofilms. Cells were constricted at the cross-walls with rounded apical cells. False branching, heterocytes, and akinetes were not observed. The complete 16S rRNA sequence (1480 pb) compared to the GenBank database showed 100% identity with *Leptolyngbya boryana* NIES-2135 (accession no. AP018203.1), *Leptolyngbya boryana* IU 594 (accession no. CP092419.1), and *Leptolyngbya boryana* IAM M-101 (accession no. AP014638.1). These data support its classification within the species *Leptolyngbya boryana*.

#### 3.1.5 *Leptolyngbya* sp. BS

The morphological description of this strain is pretty much similar to that of the previously described *Leptolyngbya* sp. R. The coding sequence for 16S rRNA (1480 pb) was compared with the GenBank database showing a 100% identity with sequences belonging to the *Leptolygnbya* genus such as *Leptolyngbya boryana* NIES-2135 (accession no. AP018206); *Leptolyngbya boryana* CCALA 1076 (accession no. OR887238), Ahmednagar, Maharashtra (India), from a sugar cane field; *Leptolyngbya foveolarum* VP1-08 (accession no. FR798945), Villa La Pietra, Firenze (Italy), from a concrete fountain with stagnant water and gray dry crust.

### 3.2 Secondary Effluent Composition

The secondary effluent had to be characterized for its nutrient content as high concentrations in it could hinder PHB production. The amount of nitrate and phosphate was low (1.17 mg N-NO_3_^–^/L and 0.57 mg P-PO_4_^3–^/L, respectively), while the concentration of ammonium was relatively high (17.67 mg N-NH_4_^+^/L), making the secondary effluent presumably not a good medium for PHB accumulation. The ammonium concentration decreased after 1 week of cultivation (Figure 3). Across all strains and independently of the acetate concentration, ammonium removal was almost totally achieved. The initial ammonium concentration of 17.67 mg/L was substantially reduced after the incubation period, with most residual values falling below 1 mg/L. In the case of *Synechocystis* sp. AP, complete removal was observed, while a few other cases resulted in slightly higher residuals between 2 and 3 mg/L; nevertheless, these concentrations are still considered low compared to the initial 17.67 mg N-NH_4_^+^/L. In certain cultures, this extensive ammonium depletion may have been linked to biomass growth, whereas in others, nitrogen removal occurred without a corresponding increase in biomass.

**Figure 3:**
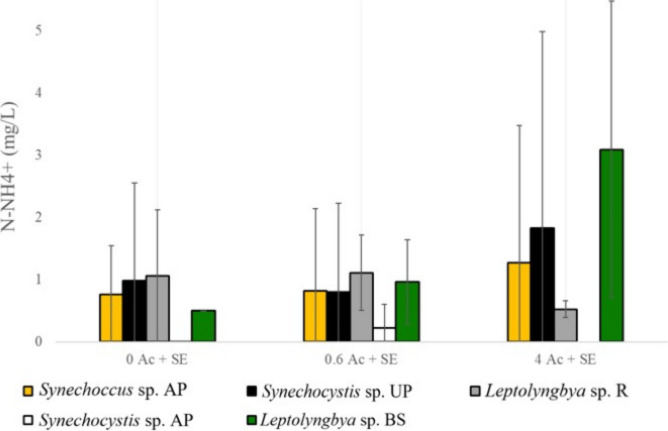
N-NH_4_^+^ final concentration after 7 days of incubation for all strains at each acetate condition in the secondary effluent. 0 Ac + SE: Secondary effluent without acetate; 0.6 Ac + SE: secondary effluent with an acetate concentration of 0.6 g/L; 4 Ac + SE: secondary effluent with an acetate concentration of 4 g/L.

The observed consumption for those in which no biomass growth was observed could be explained by NH_3_ volatilization at high pH values^37^ in which cultures were found (Figure S1, Supporting Information), or alternative pathways, such as pigment synthesis.^38^ This represents a vital mechanism whereby cells assemble components during the night for immediate use upon return of light. Nevertheless, the secondary effluent seems suitable as a medium for previous growth of cultures rather than use it for the accumulation of PHB polymers. Still, this medium was evaluated as a potential candidate for PHB production because, despite the well-established evidence that nutrient availability generally suppresses polymer synthesis,^39^ it remained uncertain whether nitrogen in the form of ammonium would have the same inhibitory effect in the absence of other nutrients. Furthermore, assessing whether PHB could still be accumulated under these conditions was of particular interest, as such a finding would have important implications for process economics. Specifically, it would suggest that cyanobacteria may be able to synthesize PHB directly from raw effluents, such as those used in this study, without requiring prior nitrogen removal pretreatment, thereby reducing production costs and simplifying downstream operations.^40^

### 3.3 pH Variation

The pH in the initial inocula ranged from 7 to 8 in the tubes with modified BG11 with no acetate and with a concentration of 0.6 g/L (Figure S1, Supporting Information). Higher pH values around 8.3 were obtained with the addition of 4 g/L acetate in modified BG11 medium. Notwithstanding, in the case of the tubes inoculated with the secondary effluent, pH values were in general higher than the ones obtained in modified BG11, ranging from 8.5 to 9 independently of the addition of acetate.

No great changes in pH were observed between the beginning of the experiment and the pH after 7 days of inoculation (Figure S1, Supporting Information). The highest change was observed in the cultures with modified BG11. In addition, it is remarkable that at the start of the test, there were differences on the pH of the media depending on the addition of acetate, whereas in the secondary effluent, the presence of acetate did not seem to influence pH. Those differences could be attributed to a higher alkalinity on the secondary effluent than in the modified BG11 medium. The latter did not have alkalinity on the grounds of being depleted of nutrients such as inorganic carbon among others. Conversely, the secondary effluent from this wastewater treatment plant is being reported to have high alkalinity levels due to its inorganic carbon concentration.^41^

### 3.4 Biomass Evolution

Biomass results after the 7-day incubation were significantly different between strains (*p*-value <0.01), despite having inoculated all of them at 0.4 gVSS/L at the beginning of the experiment (Figure 4).

**Figure 4:**
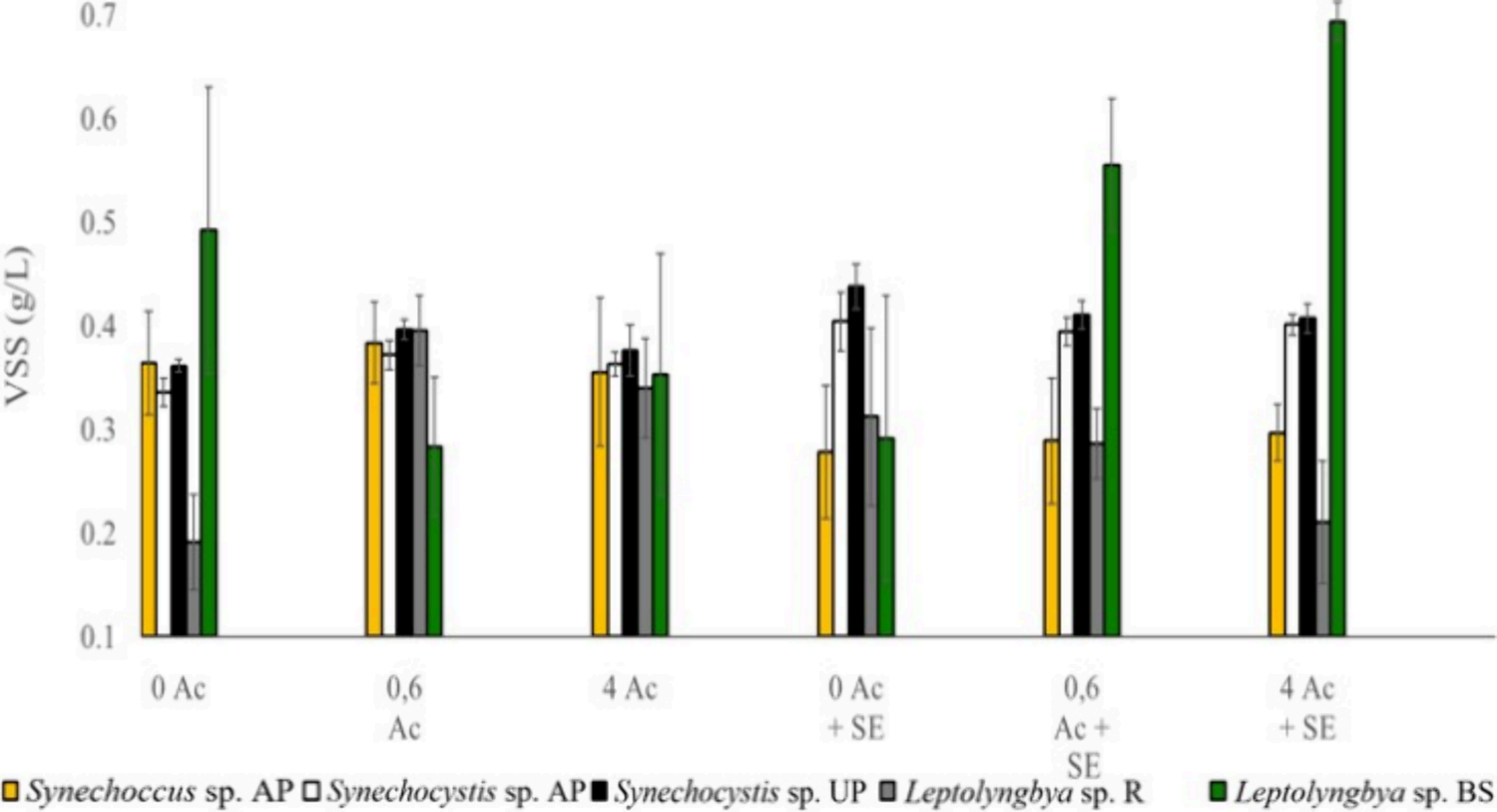
Biomass values expressed as VSS after 7 days of incubation under darkness for all unicellular strains. 0 Ac: Modified BG11 without acetate; 0.6 Ac: modified BG11 with an acetate concentration of 0.6 g/L; 4 Ac: modified BG11 with an acetate concentration of 4 g/L; 0 Ac + SE: secondary effluent without acetate; 0.6 Ac + SE: secondary effluent with an acetate concentration of 0.6 g/L; 4 Ac + SE: secondary effluent with an acetate concentration of 4 g/L.

*Synechococcus* sp. AP exhibited a clear decrease in biomass concentration after 1 week of growth in the secondary effluent. These results were significantly lower (*p*-value <0.01) than those obtained with BG11, where biomass remained stable at values close to the inoculation concentration. This decrease was evident not only from turbidity measurements but also from visual inspection, as the culture became more translucent after the 7-day incubation. Therefore, *Synechococcus* sp. AP seems not to be a good candidate for PHB production using a secondary effluent. Although *Synechococcus* sp. is widely used for nitrogen removal from wastewater^42^ and reported to grow with ammonium as a nitrogen source, concentrations of ammonium around 20 mg/L or higher have a detrimental effect on *Synechococcus* sp.^43^ Hence, the reason why populations have not thrived with a secondary effluent is that ammonium concentrations were almost at 20 mg/L.

For both *Synechocystis* sp. strains, the biomass concentration followed the same fashion (*p*-value >0.05). No significant differences were observed among the acetate concentrations applied (*p*-value >0.05). However, there were differences depending on the type of medium in which cells were inoculated (*p*-value <0.01). When using modified BG11, the VSS concentration slightly decreased from the original 0.4 gVSS/L. Meanwhile, with the secondary effluent, the biomass remained stable at around 0.4 gVSS/L after the 7-day inoculation in every condition. Those results are similar to other studies in which *Synechocystis* sp. cells are grown in the secondary effluent in which populations are even able to grow significantly in the presence of ammonium as the main nitrogen source.^41^ The main reason why *Synechocystis* sp. cells from this study have not grown might be because they were placed under dark conditions in which photosynthesis could not take place. These results stand out in contrast with those obtained by *Synechococcus* sp. AP where the kind of medium used affected oppositely, reflecting which medium would be used for each strain in a hypothetical scaling-up of the process.

Regarding filamentous strains, this parameter could not be measured due to lack of reliability between the turbidity and VSS correlation relationship. Directly measuring VSS was not possible, given that the total amount of biomass obtained after the experiment was needed for PHB measurements. Instead, the total dry biomass obtained after lyophilization was weighed, and a calculation of VSS concentration was done considering the remaining final volume. For unicellular strains, the procedure was conducted in a similar manner, apart from following the VSS methodology. However, the results displayed are the ones from correlation of turbidity since those results have more reliability and less variance between replicates.

The final biomass from *Leptolyngbya* sp. R did not show significant differences between using modified BG11 or the secondary effluent (*p*-value >0.05). Nonetheless, the results obtained using the three acetate concentrations showed different responses depending on the medium used (*p*-value <0.01). Depletion of acetate diminished the biomass concentration when using BG11, while with the secondary effluent, it remained around 0.4 gVSS/L. Conversely, high acetate concentrations diluted in BG11 made the biomass concentration hold at 0.4 gVSS/L whereas the same acetate concentrations using the secondary effluent lead to a slight decrease in biomass. Overall, it appears that this particular strain did not grow during the accumulation period, which is consistent with the expected outcome under the imposed conditions. The observed decline in biomass may be related to limited acetate uptake efficiency or to the interaction with the chemical complexity of the secondary effluent, factors that were not specifically addressed in this study. Similar strain-dependent differences in acetate assimilation have been reported for *Leptolyngbya boryana* and related filamentous cyanobacteria, where heterotrophic growth is strongly influenced by mutations or regulatory variability in carbon assimilation pathways.^44,22^ Moreover, wastewater-derived media introduce additional stressors, such as high ammonium levels, alkalinity, and organic cosubstrates, which can alter acetate metabolism and energy allocation compared to defined media like BG11.^41^ Taken together, these findings highlight that the distinct response of *Leptolyngbya* sp. R may arise from a combination of intrinsic metabolic traits and medium-dependent interactions, underscoring the need for further genomic and physiological characterization.

*Leptolyngbya* sp. BS is the strain that obtained the most different results compared to the others. The response between the acetate concentration and type of medium changed completely depending on the condition (p-value <0.01). Incubation with acetate diluted in modified BG11 helped the remaining biomass to be 0.4 gVSS/L to a certain extent, while no acetate addition seems to increase its biomass. This result was not expected since cells were not set at growing conditions. Perhaps, these filaments had internal storage that allowed cells to grow.

Contrarily, in the secondary effluent, acetate addition shows a dramatic increase in biomass whereas no acetate addition maintains biomass levels with no change from incubation. These results clearly show how this strain can grow perfectly in heterotrophic conditions and how it is able to uptake acetate for its own growth when using the secondary effluent but not with BG11. Some studies reported that several variants of *Leptolyngbya boryana* are able to grow heterotrophically depending on particular mutations on the global transcriptional regulatory systems involved in photosynthetic activity.^44^ This could be the reason behind the divergence in behavior between both *Leptolyngbya* sp. strains. Moreover, it makes sense that if that strain is able to grow heterotrophically, it only happens when acetate is added to the secondary effluent. Given that, under those conditions, cells had both organic carbon and other nutrient sources such as nitrogen and phosphorus, differently from the modified BG11 where those nutrients were depleted.

### 3.5 PHB Production

The data obtained were significantly different between strains and all conditions applied (*p*-value <0.01). Results clearly show that there is a tendency to produce more PHB as the acetate concentration was higher (Figure 5). However, for all tested strains, the secondary effluent had lower PHB production resulting in a necessary increase in acetate concentration to reach the same PHB values as the ones found in modified BG11 media at lower acetate levels. The reason behind the lower PHB yield in the secondary effluent could be due to the high initial ammonium concentration. As widely described in the literature, PHB is synthesized in starving conditions of nitrogen and phosphorus sources.^45,46^ Similar observations have been reported in other studies where excess nitrogen availability suppressed PHB accumulation in cyanobacteria, as the cells preferentially directed carbon flux toward biomass rather than storage polymers.^47^ Regarding the two strains with lower PHB results, *Synechococcus* sp. AP and *Leptolyngbya* sp. BS, no significant differences were observed between the different acetate conditions tested (*p*-value >0.05 for both strains).

**Figure 5:**
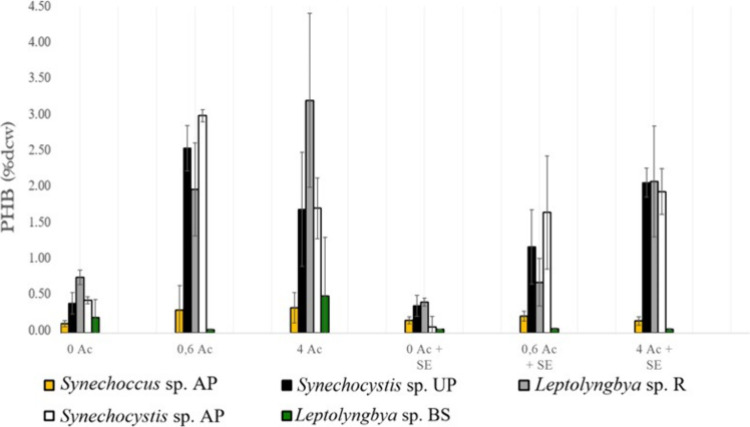
PHB results in percentage of dry cell weight (%dcw) for the five different strains tested at the six different conditions. 0 Ac: Modified BG11 without acetate; 0.6 Ac: modified BG11 with an acetate concentration of 0.6 g/L; 4 Ac: modified BG11 with an acetate concentration of 4 g/L; 0 Ac + SE: secondary effluent without acetate; 0.6 Ac + SE: secondary effluent with an acetate concentration of 0.6 g/L; 4 Ac + SE: secondary effluent with an acetate concentration of 4 g/L.

Both *Synechocystis* strains followed a similar fashion. In the case of using modified BG11, the optimal acetate concentration was around 0.6 g/L and high amounts of acetate seemed to be counterproductive. Since *Synechocystis* cells have no efficient transport mechanism for acetate uptake,^48^ high concentrations of acetate may remain on the media leading to toxicity due to depletion of proton promotion force and anion accumulation.^49^ In the case of using the secondary effluent, PHB production seems to be correlated as long as the acetate concentration increases. Thus, the response where the acetate concentration was different depending on the type of medium that was used (*p*-value <0.05) could be due to the fact that in the secondary effluent, there was a compound that counteracted the toxicity of high acetate concentration, such as its buffer capacity due to higher alkalinity as previously discussed. Notwithstanding, these parameters were not analyzed, so all discussions on this regard are merely hypothetical. This highlights the need for future studies to characterize the chemical composition of effluents in detail since cofactors such as alkalinity, trace metals, or organic compounds may significantly influence PHB yields. The best conditions for PHB production with those strains are with an acetate concentration of 0.6 g/L in modified BG11 with a maximum accumulation around 3.1% PHB in dcw. These results are similar to the ones found in another study^24^ but remain considerably lower than heterotrophic bacterial systems, where the PHB content often exceeds 60% dcw.^50^ This underlines both the limitations and the opportunities of using photoautotrophic systems for sustainable PHB production. It is expected to have higher amounts of PHB at a bigger scale after many cycles of selective conditions for PHB production as described in another study.^23^

Regarding *Leptolyngbya* sp. R, the PHB production profile appears as it would be expected; the higher the acetate concentration, the higher the PHB. This fashion is followed in both modified BG11 and the secondary effluent.

Therefore, the interaction response between the acetate concentration and type of medium was low (*p*-value >0.05).

However, results in the secondary effluent are significantly lower than the ones in modified BG11 (*p*-value <0.05). This might be due to the same reason as the aforementioned strains; the presence of ammonium hinders PHB production as cells are not under in fully starving conditions. The highest result was obtained at 4 g/L acetate in modified BG11 with around 4.1% PHB in dcw in one of the triplicates; the average, however, was around 3.2%. Despite obtaining significantly similar results to the ones from *Synechocystis* sp. strains, in the case of *Leptolyngbya* sp. R, this result was only achieved when supplementing with 4 g/L acetate, while *Synechocystis* sp. strains only required 0.6 g/L acetate. Although this result is modest, it indicates that this strain responds positively to acetate supplementation and could be optimized further under nutrient limitation strategies. This result is much lower than productions from another study in which *Leptolyngbya* sp. could reach PHB values around 32% in dcw^19^ undergoing similar cultivation conditions as this study. Notwithstanding, those high results were obtained after 22 accumulation days, while in this study, the accumulation only lasted for 1 week, suggesting that in a longer incubation, higher PHB would have been obtained. This time-dependent accumulation has been reported elsewhere, reinforcing the notion that cyanobacterial PHB synthesis is a gradual process that benefits from extended cultivation under selective pressures.^51^ Although the result of 3.2% does not have a big relevance in terms of not being enough for commercial purposes, it sheds light on which conditions are suitable for maximizing PHB and in which direction research has to be developed.

About *Synechococcus* sp. AP and *Leptolyngbya* sp. BS strains, PHB results were so low that no big differences between culturing conditions were clearly appreciated (*p*-value >0.05). It may be that these strains do not have a capacity to produce discernible amounts of PHB; therefore, no variation is obtained despite increasing the acetate content. For *Synechococcus* sp. AP, the results resemble those reported in other studies where PHB production did not exceed 1% dcw.^20^ Conversely, other investigations have shown PHB contents of up to 30% dcw in certain *Synechococcus* strains.^21^ These contrasting outcomes suggest that additional cultivation parameters must be better understood to identify the key factors that enable higher productivities. Not to mention, *Leptolyngbya* sp. BS was expected to have less percentage of PHB as with the biomass results; it seems that this strain is uptaking acetate for its own growth rather than for accumulating PHB (Figure 4).

With all these results, there is a difference in production between different strains found in nature. It was expected to obtain similar results between both *Synechocystis* strains (*p*-value >0.05) given their morphological and genetic similarity as previously described in the morphological and molecular identification of strains. Nonetheless, it was quite surprising to obtain such different results between both *Leptolyngbya* strains (*p*-value <0.01). The reason may be due to small genetic differences between them even though their genetic analysis seems to conclude that both strains belong to the same species *Leptolyngbya boryana*. This highlights that genotypic similarity does not always translate into metabolic homogeneity, as minor genetic or regulatory differences can significantly affect carbon allocation pathways and PHB accumulation potential.^52^ These results support the strategy of initiating cultures from a single cell or filament to establish a stable and homogeneous population, which may exhibit distinct behaviors compared with other cultures despite belonging to the same species. Overall, the PHB productivities obtained in this study remain lower than some of the values reported in the literature^18,19^

Nonetheless, the results provide valuable insight into which types of media are most suitable for enhancing PHB production and help to distinguish strains with the potential for further investigation from those less promising for future large-scale applications.

## 4 Conclusions

Effective isolation of cyanobacterial strains was achieved by combining traditional and complementary techniques. Among the isolates, we found that *Synechocystis* sp. AP showed the highest PHB accumulation capacity at only 0.6 g/L acetate, reaching yields comparable to strains that typically require more than 1 g/L in other studies.

*Leptolyngbya* sp. R remains a potential candidate but is limited by its dependence on elevated acetate, while *Synechococcus* sp. AP and *Leptolyngbya* sp. BS accumulated less PHB. Modified BG11 medium supported optimal production, whereas the secondary effluent was unsuitable due to an excess in ammonium. Although these results are not yet sufficient for commercial applications, they demonstrate the benefit of reducing acetate requirements and provide a clear direction for ongoing experiments aimed at optimizing PHB scale-up.

## Supplementary Material



## Notes

Table showing the location from which strains were obtained, pH data from the starting point and after 7 days of PHB incubation, and graphs showing the correlation between VSS and turbidity (g/L with respect to NTU) (PDF)
