# Supplementary material for: Assessment of Polyhydroxybutyrate Production by Cyanobacteria Strains Isolated from Environmental Water Sources Using a Secondary Effluent
Source: ACS ES T Water. 2025 Nov 7;5(12):7267–78. doi: 10.1021/acsestwater.5c00677 (PMC12707228; doi:10.1021/acsestwater.5c00677)
Supplement: Supplementary file 1 [file ew5c00677_si_001.pdf]

Supporting Information

Assessment of polyhydroxybutyrate production by cyanobacteria strains isolated from environmental water sources using secondary treated wastewater

Artai Lage[1], Ester Berrendero[2], Laura García-Abad[2], Cristina Martínez-Gutiérrez[2], Joan Garcia[3], Eva Gonzalez Flo[1]*

[1] GEMMA - Group of Environmental Engineering and Microbiology, Department of Civil and Environmental

Engineering, Escola d'Enginyeria de Barcelona Est (EEBE), Universitat Politècnica de Catalunya·BarcelonaTech, Av. Eduard Maristany 16, Building C5.1, E-08019 Barcelona, Spain.

[2] Department of Applied Biology, Universidad Miguel Hernández, Elche, 03202, Spain

[3] GEMMA - Group of Environmental Engineering and Microbiology, Department of Civil and

Environmental Engineering, Universitat Politècnica de Catalunya·BarcelonaTech, c/ Jordi Girona 1-3, Building D1, E-08034 Barcelona, Spain

Corresponding author: eva.gonzalez.flo@upc.edu

Strain	Place	Coordinates	Ref
***Synechocystis* sp. AP**	Agricultural pond	Viladecans, Spain, 41º28’78.6”N, 2º04’36.9”E).	*Synechocystis* sp. R2020
***Synechococcus* sp. AP**	Agricultural pond	Viladecans, Spain, 41º28’78.6”N, 2º04’36.9”E	*Synechococcus* sp. R2020
***Synechocystis* sp. UP**	Urban pond	Barcelona, Spain, 41°24′31.0”N 2°12′49.9″E	*Synechocystis* sp. L2023
***Leptolyngbya* sp. R**	Besòs River	Sant Adrià de Besòs, Spain, 41°25′20.2”N 2°13′38.2″E	Leptolyngbya sp. L1_2023
***Leptolyngbya* sp. BS**	Besòs River shore	Sant Adrià de Besòs, Spain, 41°25′20.2”N 2°13′38.2″E	Leptolyngbya sp. L2_2023

Table 1. Location of the samples that were used for developing the microbiome cultures from which the strains were isolated.

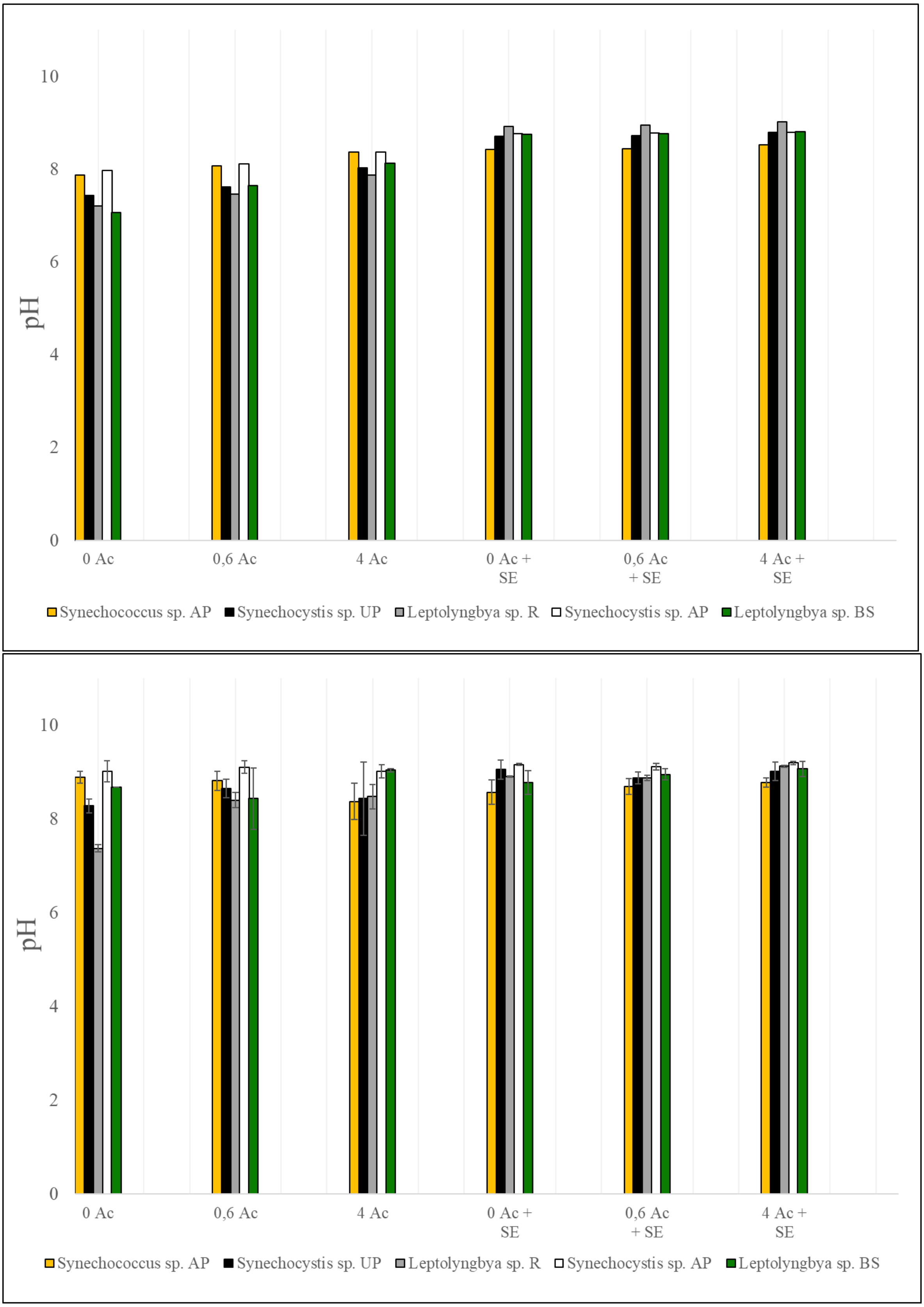

Figure 1. Graph showing the pH values for each strain at every condition at the day of the inoculation (A) and after 7 days incubation (B).

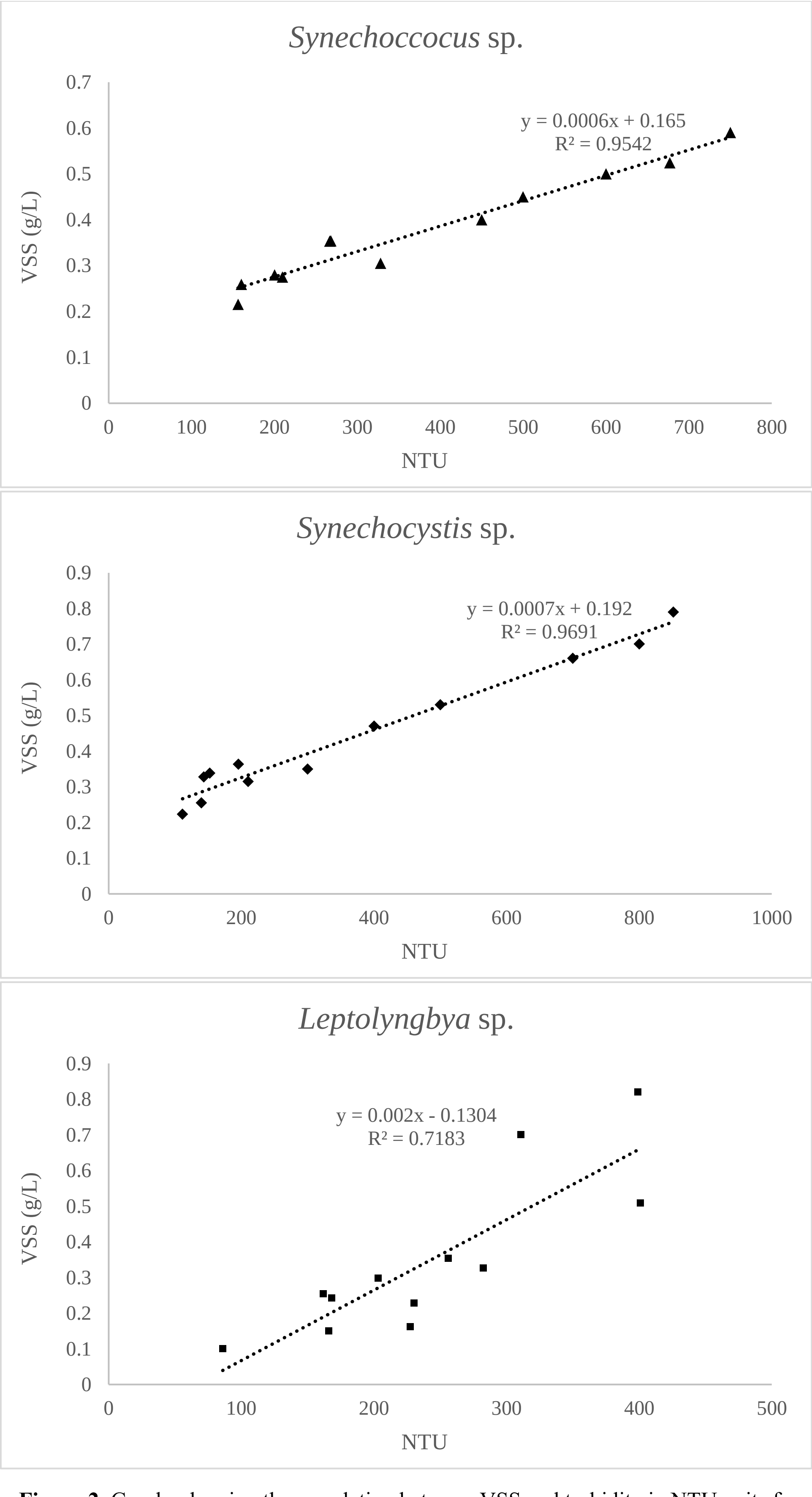

Figure 2. Graphs showing the correlation between VSS and turbidity in NTU units for *Synechococcus* sp. *Synechocystis* sp. and *Leptolyngbya* sp. strains.
